# Anti-SARS-CoV-2 nucleoprotein IgA is associated with worse disease severity in critically ill COVID-19 pneumonia patients

**DOI:** 10.3389/fimmu.2026.1870217

**Published:** 2026-07-14

**Authors:** David M. Brett-Major, Dylan S. George, Eric D. Morrell, Carmen Mikacenic, Julie M. Carstens, Laura E. Evans, Mark M. Wurfel, Pavan K. Bhatraju, M. Jana Broadhurst

**Affiliations:** 1 Department of Epidemiology, College of Public Health, University of Nebraska Medical Center, Omaha, NE, United States; 2 Division of Infectious Diseases, Department of Medicine, College of Medicine, University of Nebraska Medical Center, Omaha, NE, United States; 3 Department of Pathology, Microbiology, and Immunology, College of Medicine, University of Nebraska Medical Center, Omaha, NE, United States; 4 Division of Pulmonary Critical Care and Sleep Medicine, University of Washington Medical Center, Seattle, WA, United States; 5 Benaroya Research Institute, Virginia Mason Franciscan Health, Seattle, WA, United States

**Keywords:** COVID-19, critical care, IgA, IgG, lung injury, machine learning, neutrophil, SARS-CoV-2

## Abstract

While humoral responses to vaccination and infection have largely focused on IgG, there is an evolving literature on ways in which IgA may drive neutrophil activity in lung injury secondary to viral infections, including COVID-19. We evaluated 238 immune-naïve, critically ill patients in the first year of the COVID-19 pandemic. Employing a laboratory-developed, multiplexed antibody testing, clinical history, medical record, and machine learning, we pursued relationships between systemic IgA and disease severity. Individuals with elevated anti-N IgA had 4-fold odds of more severe disease. This work emphasizes the importance of continued work to understand how different elements of the immune system participate together in contributing to patient outcomes.

## Introduction

Academic medical centers and the Society for Critical Care Medicine established cooperative cohort studies to allow in-depth assessment of severe acute respiratory infection (SARI) patients. This work has linked proteomic biomarkers with severe novel coronavirus disease (COVID-19); discerned relationships between the course of the pandemic and health system strain; and, afforded a look at ways in which pandemic periods have differed from each other (1–5). These studies enrolled individuals with severe COVID-19 pneumonia in a prospective, observational cohort study to assess patient risks, allowing observation of COVID-19 disease experiences from the start of the US COVID-19 pandemic. Consequently, this work allows observations to be made in the context of a novel threat.

The presence of antibodies against SARS-CoV-2—particularly neutralizing antibodies—reduces the likelihood of severe disease (6). At the same time, antibody-mediated immunopathology may increase COVID-19 disease severity through Fc-mediated proinflammatory pathways (7). This literature has primarily focused on anti-RBD IgG, both because those antibodies predominate neutralizing antibody populations against SARS-Co-2, and they are the generative target of current vaccine strategies (8). The roles of anti-Nucleoprotein (N) antibodies and that of IgA are less well characterized, even as potential roles in immunopathology have been identified, such as IgA-mediated neutrophil activation (9).

IgA may potentiate neutrophils associated with lung injury. Interaction of IgA immune complexes with FcαR1 on neutrophils leads to the release of neutrophil extracellular traps in patients with severe COVID-19 and influenza (10, 11). Recently, research into the pathophysiology of severe dengue disease—which also has a lung injury component—demonstrates that anti-NS1 protein IgA enhances neutrophil activation in samples from patients with dengue hemorrhagic fever or shock syndrome (12). Other IgA actions include inhibiting surfactant proteins through host:virus epitope homology (autoantibodies) (13), and thromboembolic complications linked to glycosylated IgA (14). Yet, IgA in bronchoalveolar lavage fluid has been associated with decreased COVID-19 mortality (15). Answering outstanding questions regarding the role of IgA in severe COVID-19 pneumonia may lead to therapeutic strategies which can augment current approaches and favorably impact mortality. In a multi-site, randomized, placebo controlled trial assessing antiviral (remdesivir) and targeted anti-inflammatory (baricitinib) therapies under advanced systems of care in patients with moderate to severe COVID-19 pneumonia, mortality among patients receiving optimal interventions remained 5 percent (16). In patients requiring mechanical ventilation or extracorporeal membranous oxygenation at admission, it was over 10 percent.

Using a laboratory-developed immunoassay against multiple SARS-CoV-2 antigens, we profiled antibody responses and host factors in a large cohort of the earliest critically-ill COVID-19 SARI patients, demonstrating the independent contribution of anti-nucleoprotein (N) IgA to the severity of COVID-19 lung disease.

## Methods

### Study design and participant characterization

COVID-19 patients admitted to the intensive care unit (ICU) at the University of Washington Medical Center, Seattle, in the first year of the COVID-19 pandemic (April, 2020, through May, 2021) were enrolled in the Severe Acute Respiratory Infection – Preparedness (SARI-PREP) study (UNMC single IRB No. 544-20-FB; UW IRB No. 9763; NCT04786301). Demographic information, past medical history, oxygen and other interventional requirements, as well as APACHE II scoring information were collected on each enrolled patient (17). NIH Ordinal score (scored 1 through 8) at time of intake was the primary outcome (18). Factors relevant to risk for severe COVID-19 pneumonia were selected for incorporation in into our analytic workflows based upon our previous work exploring heterogeneity and risk of bias in mortality analyses of COVID-19. These included age 65 years or older; days of illness ≥14 days; body mass index (BMI) ≥30 kg/m^2^; and, a past medical history of chronic obstructive pulmonary disease (COPD) (19). SARS-CoV-2 sequencing was performed at the University of Washington as part of statewide public health response (20).

### Laboratory-developed immunoassay

An in-house, multiplexed, bead-based immunoassay was developed for the quantitation of anti-SARS-CoV-2 IgG and IgA antibodies. SARS-CoV-2 protein antigens included canonical Spike receptor binding domain (RBD, expressed in HEK293 cells; Sino Biological, Ltd., 40592-V08H1) and N (fusion protein with MBP linker expressed in *E. coli*; UNMC Structural Biology Facility, accession: YP_009724397.2). Assay buffers included wash buffer (1X PBS-TBN with 0.05% Tween, 1% BSA, 0.1% Sodium Azide; Teknova, P0220) and activation buffer (0.1M NaH2PO4; Sigma-Aldrich, 7558-80-7) dissolved in MilliQ water. Magnetic microsphere beads with unique fluorescent signatures (Luminex Corp) were coupled to protein antigens (80 pmol protein/million beads) following activation of surface carboxyl groups. Beads were washed and counted for storage at 2 million beads/mL. Diluted beads were stored as single-plex solutions protected from light at 4 °C. Secondary antibodies included R-Phycoerythrin-conjugated goat anti-human IgG, IgA, and IgM (Jackson ImmunoResearch, 109-115-170, 109-115-011, and 109-116-129, respectively). All secondary antibodies were diluted 1:100 in assay buffer prior to secondary incubation.

### Antibody measurements

Sera were collected within three days of presenting to the ICU and stored at -80 °C until analysis. Thawed sera were diluted 1:100 in assay buffer. 50uL of diluted sample was mixed with the multiplexed bead mix at 1,250 beads per reaction. Primary incubation was performed in a 96-well plate covered in foil on a BioShake IQ Thermomixer (Bulldog Bio, Inc.) at 37 °C, 600rpm, for 30 minutes. Following primary incubation, the 96-well plate was placed on a magnet and the paramagnetic beads were allowed to bind for 2 minutes before the supernatant was removed by vertical suspension. 200ul of assay buffer was added to each reaction well, then the plate was placed back on the Bioshake, uncovered, for 2 minutes, shaking at 600rpm, at 37 °C. This wash procedure was repeated twice between primary and secondary incubation. 100ul of diluted detection antibody mix was added following the final removal of the wash buffer. This detection mix was also allowed to shake at 600rpm, 37 °C, for 30 minutes, covered in foil. Following secondary incubation, the plate was washed twice and beads were resuspended in 100uL of assay buffer and allowed to shake, covered on the Bioshake at 600rpm and 37 °C for 10 minutes prior to reading on the Luminex IntelliFlex instrument for measurement of median fluorescent intensity (MFI) using the dual-channel setting. On each assay run, non-specific binding of secondary antibodies to protein-coupled beads was measured in wells without sample added (blank wells). MFI signal from the blank wells for each antigen target was subtracted from the sample wells for a final MFI value.

### Machine learning and statistical analyses

Microsoft Excel and R Studio version 4.4.2 were used for data handling and analysis. Descriptive analytics, ordinal and binary logistic regressions, and gradient boosting machine learning were performed. Machine learning incorporated K-fold cross-validation and hyperparametric tuning to < 10% train| test-log-loss difference. To translate relative influence into odds for the strongest linear factors, SHAP (SHapley Additive exPlanations) values and direction of influence were used to create binary breakpoints in revisited regression models. Breakpoints were selected based upon population grouping (clustering) of observations at different levels of influence (SHAP values) as indicated on the y-axis of the SHAP curves, and adjusted at boundaries for twice a conservative estimate of precision for the assay by analyte. The dataset and R code are available at https://doi.org/10.7910/DVN/UKPNLM.

## Results

Between April, 2020, through May, 2021, 241 individuals contributed 762 specimens, including women (32%), and those identifying as Hispanic (29%) and Black (14%). Half of participants presented via an outside hospital. At admission to the ICU, mean APACHE II scores were 23 (standard deviation 10) and NIH Ordinal scores indicating COVID-19 pneumonia disease severity ranged from 4 to 7. Ninety-one participants (38%) died in-hospital. Half of the participants presented before December 2020, including periods without targeted medical countermeasures. Virus sequences were predominantly B1 variants—of 57 resolvable sequences, 50 were B1, 3 A1, 4 P1. Of 241 participants, 238 had data for analysis.

### Anti-SARS-CoV-2 antibody levels in the cohort

**Figure 1:**
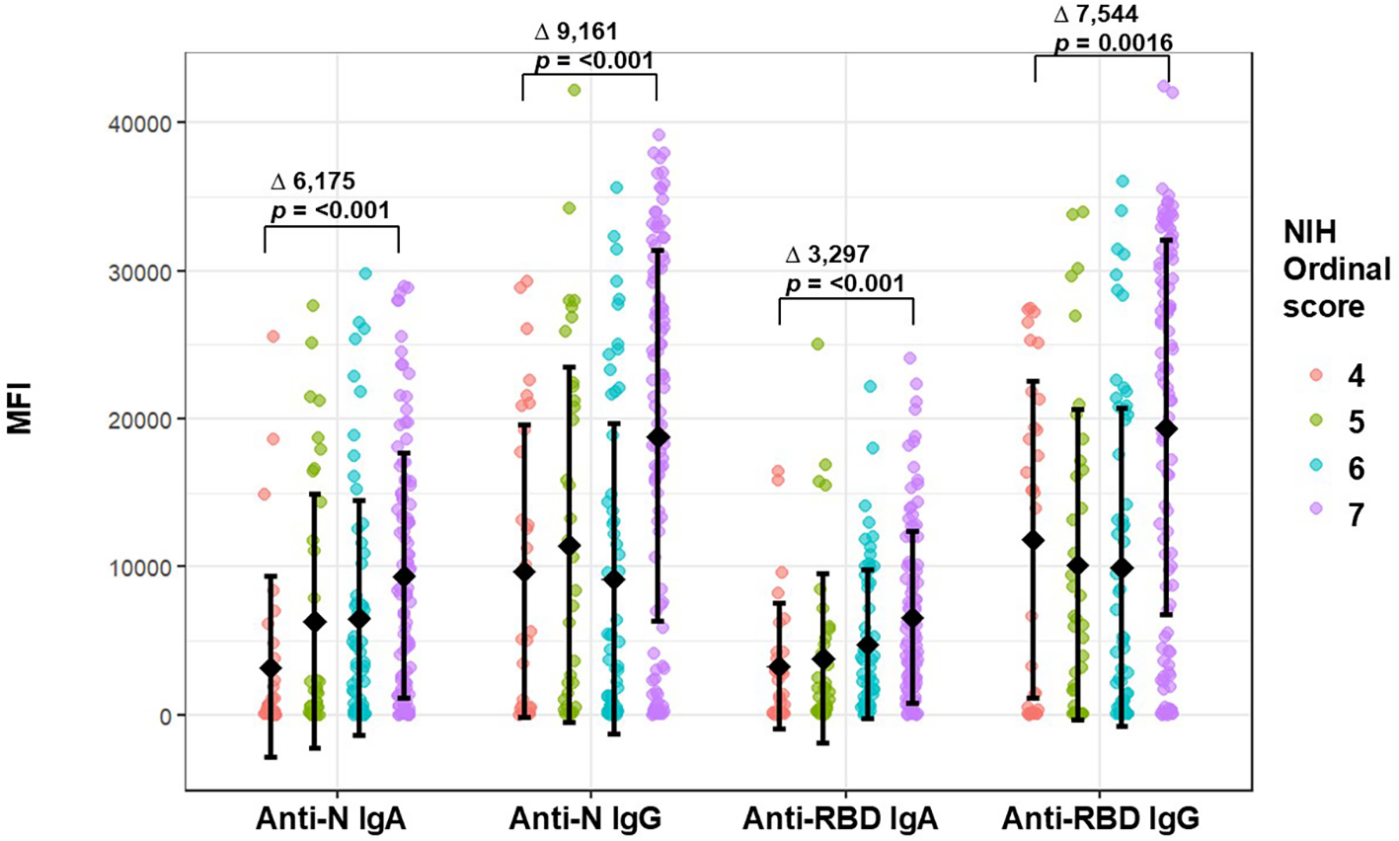
Antibody levels and common severe COVID-19 risk factors by NIH Ordinal score. Anti-SARS-CoV-2 antibody levels by disease severity. MFI by target and isotype against NIH Ordinal score at intake is shown. The difference in mean MFI with *t*-test between an ordinal score of 7 vs. 4 is shown for each combination; all four comparisons and the stepwise increase of Anti-N IgA yielded a *p* < 0.001 by Kruskal-Wallis test. NIH Ordinal scores represented are 4: hospitalized but no supplemental oxygen; 5: on supplemental oxygen; 6: on non-invasive ventilation or high flow oxygen; 7: mechanically ventilated.

Antibody results and host risk factors for severe COVID-19 pneumonia were explored for distinct relationships with the outcome. Patients with ordinal scores of 7 (the highest severity observed in our cohort) had the highest MFI values for anti-SARS-CoV-2 antibodies. (**Figure 1**, *p* < 0.001 for each combination by Kruskal-Wallis test). Anti-N IgA and anti-RBD IgA also showed consistent elevations in midrange ordinal scores in contrast to a score of 4; this reached statistical significance for anti-N IgA in the 5 vs. 4 comparison. (**Figure 1**; **Supplementary Table 1**).

**Supplementary Figure 1** shows relationships between individual host co-factors and MFI values. Those participants aged 65 years or older had lower mean MFI values across all targets than younger participants, at times reduced by more than a third of the younger participants’ mean values, including anti-N IgA with mean MFI lower by 3,015 (*p* < 0.001 by t-test). Only the difference in anti-RBD IgA did not reach statistical significance. Participants with COPD also had lower MFI values across all targets, however none of these differences were statistically significant. Mild increases in MFI values across targets were present among participants with elevated BMI, reaching statistical significance for anti-RBD IgA. The 14-days of illness breakpoint showed marked increases in IgG (reaching mean differences of approximately 10,000 MFI, each) but not IgA. The fraction of those aged 65 years or older and those who presented ≥14 days into their illness substantially differed by NIH ordinal score, while COPD and categorical BMI status did not. (**Supplementary Table 2**).

In **Supplementary Figure 2**, humoral targets are compared to each other in order to explore their differential value both overall and when considering specific host factors. Log transforms of MFI demonstrated good correlation between anti-N and RBD IgG (Spearman rho 0.82, *p* < 0.001); moderate correlation between anti-N and RBD IgA; and, moderate correlations between IgG and IgA in both targets (Spearman rho 0.58-0.65, each *p* < 0.001).

### Selection and preparation of variables for entry into machine learning

We proceeded to a multivariate analysis to inform subsequent variable formats for machine learning. This began with ordinal regression analyses on incrementally increasing NIH ordinal score. Consistent with the variability of mean MFI values among ordinal scores of 4-6 (**Figure 1**), no MFI association was observed. Only age and days of illness had statistically significant effects in the regression model. Results then were assessed for change when shifting ≥14 days of illness from a predictor to a stratifying variable, and replacing IgG and IgA to residual factors accounting for the other. This did not change the output. (**Supplementary Table 3**) and reinforced the possibility of independent effects by IgG and IgA. A binary logistic regression was then used to assess the subset of individuals who presented with the lowest (4) or the highest (7) observed NIH ordinal score, employing the four antibodies as separate predictors. Only the anti-N IgA coefficient was statistically significant, *p* = 0.036. Compositely the model produced an AUC = 0.8 (95% CI 0.72-0.88) with reasonable prediction (Hosmer-Lemeshow *p* = 0.47) and mild multi-collinearity (VIF of 1-3; scaled Brier 0.2) (**Supplementary Figure 3**).

### Associations of variables with COVID-19 severity by NIH ordinal score

**Figure 2:**
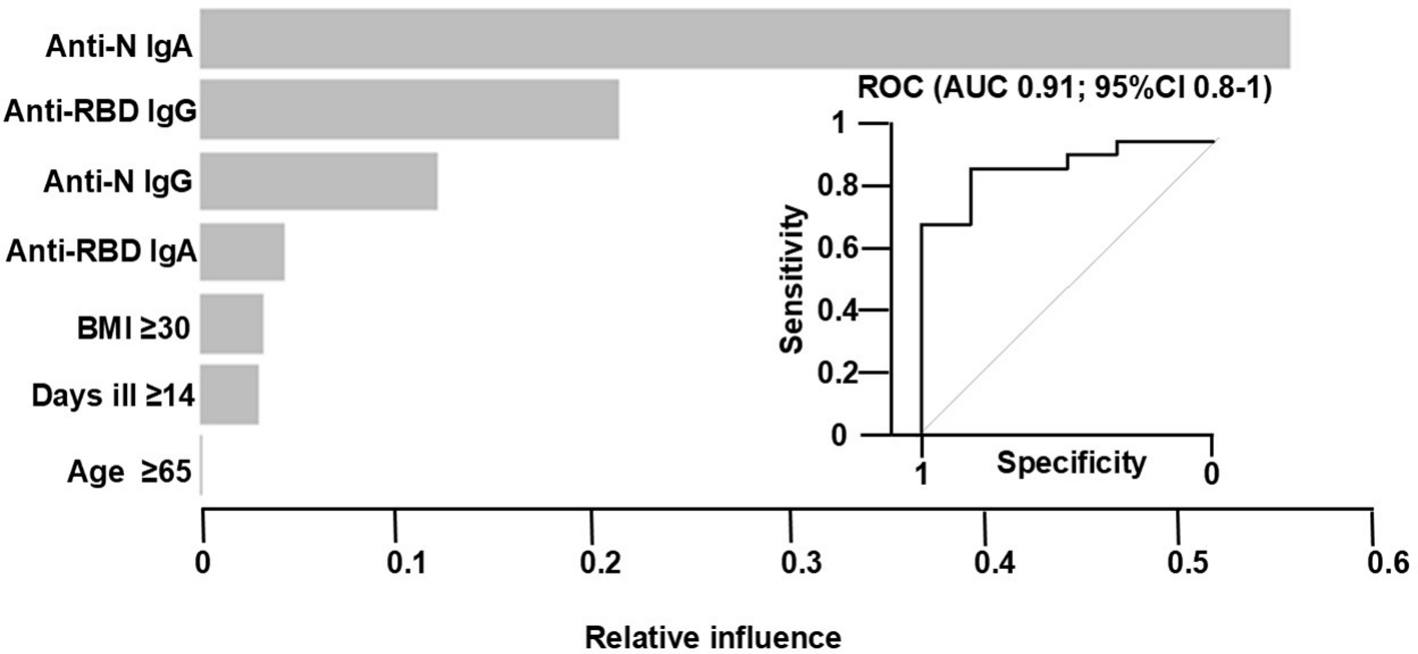
Anti-N IgA has the greatest influence on disease severity. Machine learning indicates anti-N IgA is a dominant distinguishing risk factor for severe COVID-19 pneumonia in this cohort. Relative influence output from a gradient boosting machine learning model demonstrates anti-N IgA as the leading predictor in this construct; the inset shows its receiver operator characteristic curve. COPD status did not impact the model. The train-test difference at its best iteration was 0.17, suggesting that the model was not overfitted.

These refined variables were then examined for their influence on disease severity using a gradient boosting machine learning model. The outputs elaborated the importance of anti-N IgA which had a relative influence nearly three-fold that of the next highest major factor, anti-RBD IgG. Model performance was very high, with an ROC AUC of 0.91 (95% CI 0.8-1; test-train difference of 0.16) (**Figure 2**).

Gradient boosting was repeated employing days ill as a continuous variable for quantitative bias analysis of the impact of the 14-day breakpoint. The resulting model had a mild AUC decrement and lower risk of over-fitting (0.85, 95% CI 0.65-1; test-train difference of 0.11), raising days ill to the lead influencing predictor. However, anti-N IgA and anti-RBD IgG remained strong influencers and the greater importance of anti-N IgA was preserved (**Supplementary Figure 4**).

**Figure 3:**
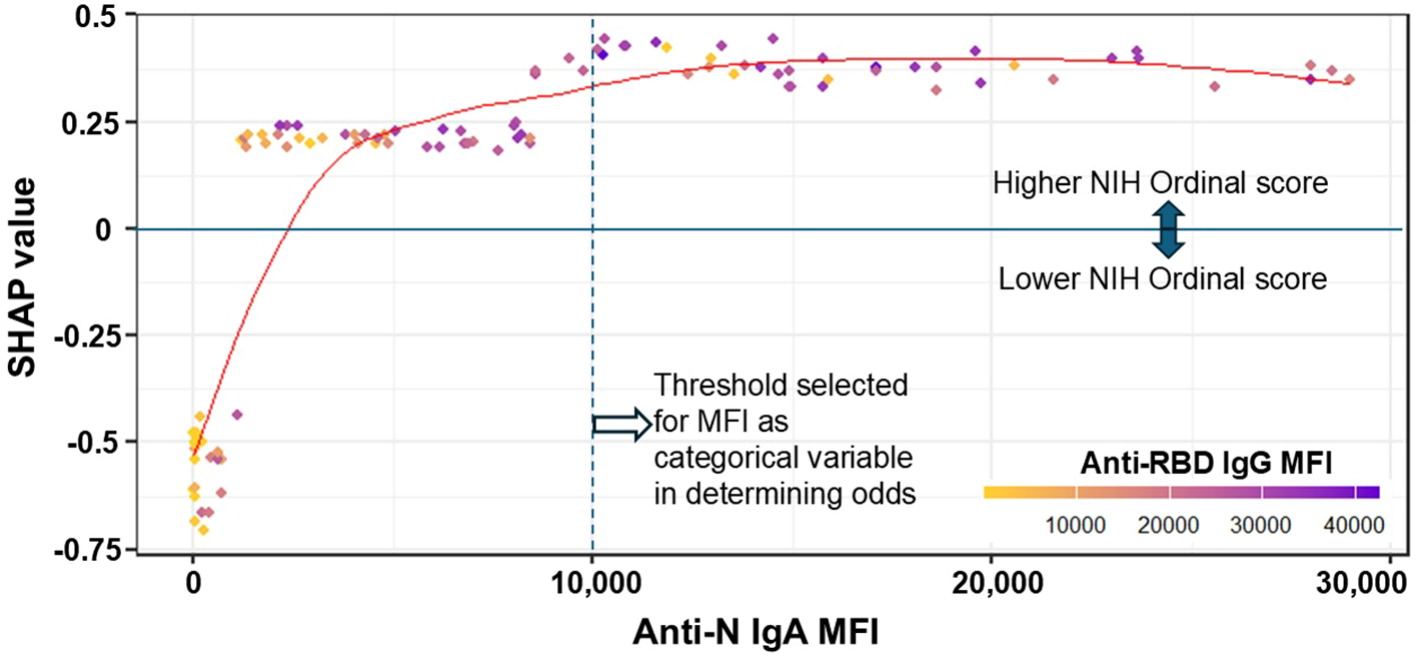
Breakpoint selection for categorical transformation of Anti-N IgA MFI. The dynamics of anti-SARS-CoV-2 antibody influence differ between anti-N IgA and anti-RBD IgG. The SHAP curve allows breakpoint determination of when the linear variable has its greatest influences in the model. This informs exploration of relationships (e.g., linear, non-parametric, sub-populations of effect) as well as binary cut-offs employed in follow-on logistic regression, allowing threshold odds ratios. The color coding reflects the magnitude of anti-RBD IgG MFI, the other leading antibody predictor, for each participant. The solid arrows pointing above and below the x-axis illustrate the areas where the data points influence the machine learning model towards a higher (up) or lower (down) NIH Ordinal score, indicating worsening disease severity. The open arrow pointing towards the right of the figure show the MFI values informing selection of the MFI breakpoints (dotted line).

**Figure 4:**
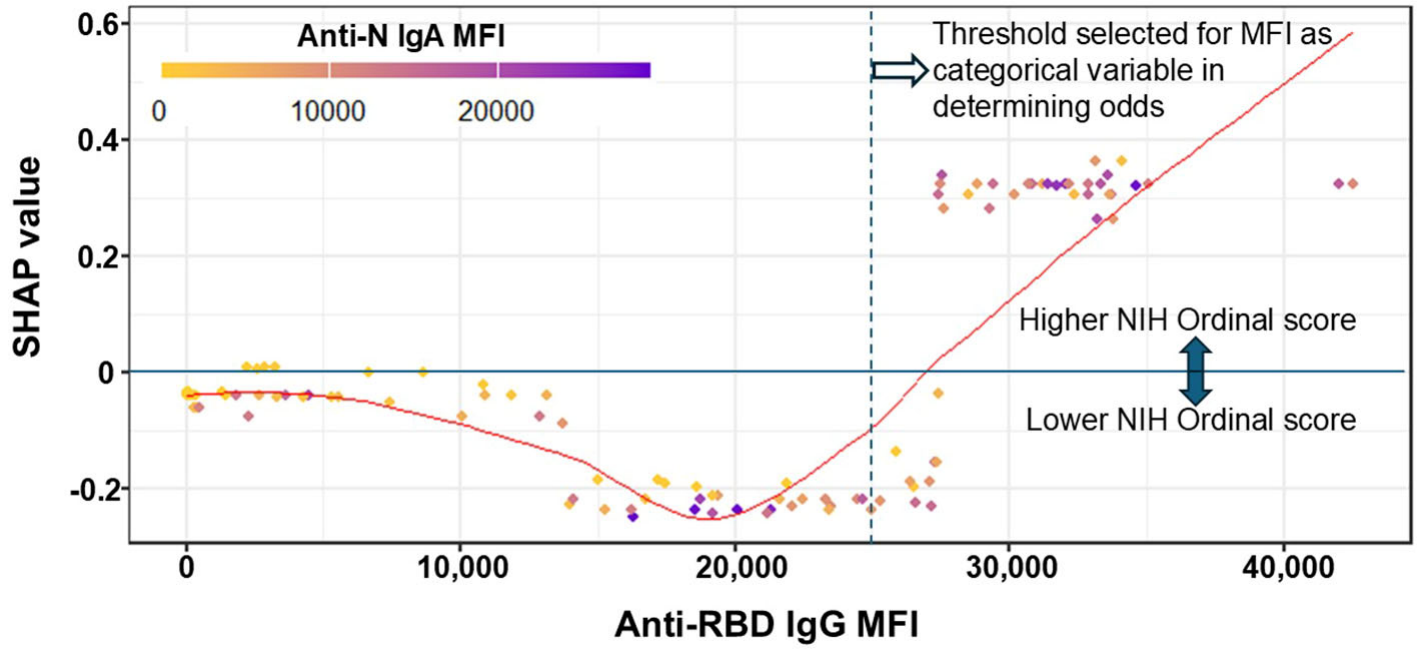
Breakpoint selection for categorical transformation of Anti-RBD IgG MFI. Similar to **Figure 3**, here, the SHAP curve for Anti-RBD IgG MFI is depicted in relation to the NIH Ordinal score, with participant-level color coding for the corresponding Anti-N IgA MFI.

To aid in contextualizing the machine learning result by generating odds, we used the outputs from the first machine learning model (days ill as a categorical variable; **Figure 2**) and selected SHAP-informed, conservative breakpoints of 10,000 MFI for anti-N IgA (**Figure 3**) and 25,000 for anti-RBD IgG (**Figure 4**) for binary logistic regression comparing ordinal scores of 4 and 7. In this model, only anti-N IgA’s effect reached statistical significance (OR 4.3, 95% CI 1.2-20; AUC 0.81, 95% CI 0.73-0.89); Hosmer-Lemeshow *p* = 0.74; scaled Brier 0.19). Subsequent addition of an interaction term for the binned anti-N IgA and anti-RBD IgG showed no effect, again indicating anti-N IgA’s independent role. Next, we performed a quantitative bias analysis testing the impact of different anti-N IgA and anti-RBD IgG threshold values used to bin results on the regression model’s performance and outputs. (**Supplementary Table 4**) Performance of the model as well as odds ratios associated with age, BMI, and COPD remained stable when increasing and decreasing each threshold by 5,000 MFI while keeping the other value at its original set point. The Anti-N IgA associated odds ratio was unchanged with a decrease in anti-RBD IgG to 20,000 MFI. Its estimate of effect was mildly decreased with a loss of statistical significance with an anti-RBD IgG threshold increase to 30,000 MFI (OR 3.4, 95% CI 1-16.2). The anti-RBD IgG, anti-N IgG, and anti-RBD IgA associated odds remained at approximately 1 through-out the quantitative bias analysis, except when using a threshold of 30,000 MFI for anti-RBD IgG in which case the result for that analyte was uninterpretable.

## Discussion

We show that virus-specific IgA is associated with severe COVID-19 pneumonia in a large cohort of immune naïve, critically ill patients who predated the era of vaccination, serial infection, and broad use of antiviral and immunomodulatory therapies. These enrollments pre-dated availability of oral anti-SARS-CoV-2 therapy. The first monoclonal antibody for prevention was approved in November, 2020, and had not been given to our participants (21). Thus, these findings are particularly applicable to novel threats and have practical implications.

Our design focused on lung injury through assignment of the NIH Ordinal score as the primary outcome, reflecting COVID-19 pneumonia severity. This patient-centered scoring system addresses outcomes rather than pathophysiologic pathways. It includes all cause (hospitalization, death) and pulmonary compartment-specific aspects (need for oxygenation, positive pressure respiration, or mechanical ventilation). The score was developed for the purpose of comparability in outcomes from investigational therapeutic clinical trials in COVID-19 (18). In our descriptive analyses, comparisons of antibody levels reveal clear differences when comparing scores 4 (hospitalized without supplemental oxygen; the lowest score in our cohort) and 7 (mechanical ventilation or extracorporeal membrane oxygenation (ECMO); the highest score in our cohort), but more variable results when comparing stepwise changes with intermediate outcomes, scores 5 (supplemental oxygen) or 6 (non-invasive ventilation or high flow oxygen). This may be due to several influences including inter-provider thresholds for applying respiratory interventions as well as differences in how individual patients respond to pulmonary support when experiencing the same syndrome. Pre-existing pulmonary status, direct consequences of positive pressure ventilatory support in lung parenchyma, fluid handling from cardiac and renal function, as well as differences in fluid administration are among the influences that impact the respiratory support required by patients with lung injury even in settings that employ well protocolized critical care practices (22).

Immunologic mechanisms contributing to lung injury are diverse. They include direct responses to pathogen-mediated injury, immune complex-mediated pathways and interactions with neutrophils, non-specific systemic inflammatory cascades, and potentially trained immunity of innate responses connected to fibrosis (7, 9, 23). Relationships between anti-SARS-CoV-2 IgG and severe disease have been observed in the COVID-19 pandemic. One group showed that a composite IgG antibody result against spike protein and nucleoprotein was associated with more severe disease (24). Another study showed that earlier seroconversion to IgG and IgA against spike protein but not nucleoprotein was linked to acute respiratory distress syndrome (25). Our findings of a dominant IgA signal related to patient-centered outcome together with the evolving literature on NETosis are an example of the importance of exploring combinatorial effects of multiple immune pathways.

From a causal inference perspective, risk factors may have overlapping effects, and incorporation of recognized host factors associated with severe COVID-19 pneumonia is a strength of our analysis. Our choice of gradient boosting machine learning following stepwise regression was driven by the potential for small effect sizes as well as the need to account for multiple starting states. For instance, our approach explored whether or not it was important that patients be viewed first as having a particular IgG value who also have a particular IgA value, or vice versa. This enabled discernment of anti-N IgA’s independent effects.

We observed earlier anti-SARS-CoV-2 IgA than IgG peaks relevant to analyses incorporating duration of illness. A small longitudinal study of 11 COVID-19 patients observed earlier IgA peaking and broader repertoire among patients who experienced severe rather than mild disease (26). In our critical care cohort, higher severity patients outnumbered more mildly ill patients (those with NIH Ordinal scores of 7 and 4, respectively), skewing our observations towards this potential effect. An *ex vivo* analysis of serial B-cells from healthy adults vaccinated with an mRNA vaccine also demonstrated earlier class switching to IgA1 than both IgG1 and 2 (27). While this might indicate a post-infection, pre-illness IgA-related pathway relevant to disease severity, extrapolating such data directly to our analysis of clinical samples from ill patients could be confounded by duration of illness, as well as moderated by mode of antigen exposure (mucosal vs. injected) and other influences on inflammation. The magnitude of IgG was consistently higher than IgA results, which also can influence the time lag to achieve peak values. The literature referenced here consistently demonstrates higher magnitude IgG than IgA responses in blood following antigenic exposure from both vaccination and infection. This may in part be due to IgA1 to 2 switching as the immune response evolves (27), though this also is consistent with blood compartment total isotype loads.

Important avenues of investigation of IgA effects include population differences. Advanced age is associated with lower antibody responses, and we observed an association between age 65 years or older with lower antibody levels against three of our four targets (the lower mean MFI difference for anti-RBD IgA did not reach statistical significance). In a study seeking to intervene on immunosenescence in the setting of vaccination, younger and older adults were vaccinated with inactivated influenza vaccine (28). Hemagglutination inhibition titers but not immune activation were lower among those 60 years of age or older compared to those 35 years or younger; and, baseline immune activation was associated with lower antibody levels. Atherosclerosis is associated with immune activation and may be a pervasive confounder in assessments of immunologic responses among older adults (29). Lifetime exposure to circulating human coronaviruses have been shown to increase the anti-SARS-CoV-2 IgG response (though with less neutralization), rather than decrease them (30). In contrast, serial SARS-CoV-2 antigen exposures yield lower serum IgA responses (31). The impact of these variances among older adults is not clear. In our cohort, those aged 65 years or older were less likely to have the highest observed NIH Ordinal score of 7. We believe that this is a result of concomitant indications for critical care unit admission among older adults from comorbidities diluting the enrollment pool, rather than a biological effect as age is an established risk factor for poor outcome from severe acute respiratory illnesses, including COVID-19.

We observed a mild elevation in anti-RBD IgA among those with elevated body mass index (BMI), as well as greater disease severity. In studies of international COVID-19 vaccine products with adenoviral platforms and inactivated virus, elevated BMI was associated with lower prevalence of anti-RBD IgG response (32). In contrast, analogous analyses of mRNA vaccination and bodyweight which included did not see this effect in IgG, however IgA was mildly higher in obesity (33, 34). Obesity is an independent risk factor for severe disease, so this may relate to how that manifests.

In our study, the presence of COPD had little influence on the analytic model and overall had lower antibody levels across all four targets, though only reaching statistical significance for anti-RBD IgG and IgA which may be a consequence of statistical power. COPD patients without acute disease have been observed to have more total IgA than controls at baseline, as well as anti-cytokeratin IgA (35, 36). This chronic immune activation may blunt epitope-specific responses and yet be relevant to the IgA-neutrophil pathway of interest here; however, recent data suggests that the propensity for pulmonary dysbiosis in COPD may be closely associated with compartmental and systemic antibody responses (37). Our cohort may also have been affected by COPD patients’ triggers based upon their underlying lung disease for escalated care that may not always relate to their COVID-19. In future prospective and interventional studies employing humoral biomarkers, COPD status should be a stratifying variable.

Broader characterization of less explored humoral immunopathologic pathways such as those informed by this study may lead to improvements in therapies for SARS-CoV-2 and other infectious diseases patients. Immunomodulatory therapies for severe COVID-19 are important, though mortality among those who carry their indications remains high. They have primarily relied upon interruption of cascades affected by IgG (38). Inhibitors of C5a have joined therapeutic repertoires, interrupting IgA-relevant neutrophil activation pathways (39). In the case of COVID-19 pneumonia, these products could be assessed in a setting where a patient’s anti-N IgA level is used as both a gate for administration as well as a stratifying variable to assess outcomes in their use. In this way, inexpensive, accurate, and high-throughput-capable humoral profiling may contribute to effective therapeutic strategies. Both sepsis and trauma lead to lung injury, so such approaches may have very broad applications in improving case management once the relevant epitopes are understood.

This work is subject to limitations. Enrollment was restricted to critical care, limiting extrapolation to pre-hospital and pre-ICU risk. Variable lag times between onset of infection and presentation; could have led to misclassification bias risk especially for those presenting at an outside hospital. Pre-ICU care practices likely evolved over enrollment, though changes in their essential therapeutic toolkit were limited. Unrecognized vaccination may have occurred for a small number of participants enrolled when it was available. Anti-N IgA’s influence on disease severity in our model was robust; whether it exceeds anti-RBD IgG’s influence may be patient specific and anti-RBD IgG levels may be more closely tied to other risk factors. Successive checks for interaction did not show substantive IgG and IgA relationships in our models. However, in our quantitative bias analysis, the estimate of effect of anti-N IgA’s associated odds ratio for severe disease remained high though lost statistical significance when employing the highest linear range anti-RBD IgG value for our assay (30,000 MFI) as a threshold; and, it remained stable at a lower threshold (20,000 MFI). This strengthens the idea of an independent action by anti-N IgA in critically ill patients across a range of antibody values. There is a possibility of mild confounding at the highest levels of anti-RBD IgG, which could be a result of statistical power, disease pathway convergence, or an inability to discern differential pathophysiologic effects among those with the most severe disease.

Anti-N IgA is independently associated with more severe COVID-19 in immune naïve, critically ill patients. In our analyses, it had consistently stronger effects and statistical significance than anti-RBD IgG, anti-N IgG, anti-RBD IgA, selected comorbidities, and days of illness. Further study is required to better elucidate the mechanisms and incremental consequences of its actions, particularly in the context of novel threats.

## Data Availability

The datasets presented in this study can be found in online repositories. The names of the repository/repositories and accession number(s) can be found below: https://doi.org/10.7910/DVN/UKPNLM.
