# Supplementary material for: Anti-SARS-CoV-2 nucleoprotein IgA is associated with worse disease severity in critically ill COVID-19 pneumonia patients
Source: Front Immunol. 2026 Jul 14;17:1870217. doi: 10.3389/fimmu.2026.1870217 (PMC13407393; doi:10.3389/fimmu.2026.1870217)
Supplement: Supplementary file 1 [file DataSheet1.pdf]

Supplement for:

Anti-SARS-CoV-2 nucleoprotein IgA may worsen disease severity in critically ill COVID-19 pneumonia patients

Brett-Major DM[1,2,*], George DS[3], Morrell ED[4], Mikacenic C[5], Carstens JM[3], Evans LE[4], Wurfel MM[4], Bhatraju PK[4], Broadhurst MJ[3]

[1]Department of Epidemiology, College of Public Health, University of Nebraska Medical Center, Omaha NE USA
[2] Division of Infectious Diseases, Department of Medicine, College of Medicine, University of Nebraska Medical Center, Omaha NE USA
[3] Department of Pathology, Microbiology, and Immunology, College of Medicine, University of Nebraska Medical Center, Omaha NE USA
[4]Division of Pulmonary Critical Care and Sleep Medicine, University of Washington Medical Center, Seattle WA USA
[5] Benaroya Research Institute, Virginia Mason Franciscan Health, Seattle WA USA

*Corresponding author. 984395 Nebraska Medical Center, Omaha, NE 68198-4395. david.brettmajor@unmc.edu

Contents

Acronyms employed in the manuscript and supplement 3

Supplementary Table 1. Differences between antibody mean MFI for each NIH Ordinal score compared to those with a score of 4 4

Supplementary Figure 1. Bivariate relationships of co-factors with anti-SARS-CoV-2 MFI by target and isotype 5

A. Comparisons by age 65 years of age or older, or less than 65 years of age 5

B. Comparisons by whether or not the patient presented with a history of chronic pulmonary obstructive disease (COPD) 6

C. Comparisons by a body mass index (BMI) of 30 or higher, or less than 30 7

D. Comparisons by presentation at 14 days of illness or later, or presenting within 14 days 8

Supplementary Figure 2. Bivariate distributions of log-transformed humoral targets coded by NIH ordinal score and host factors 9

A. Comparisons of humoral targets, with color coding by NIH Ordinal Score 9

B. Comparisons of humoral targets, with color coding by age of 65 years of older, or less than 65 years 10

Comparison of humoral targets, with color coding by whether or not the patient presented with a history of chronic pulmonary obstructive disease (COPD) 11

C. Comparison of humoral targets, with color coding by a body mass index (BMI) of 30 or higher, or less than 30 12

D. Comparison of humoral targets, with color coding by presentation at 14 days of illness or later, or presenting within 14 days 13

Supplementary Table 2. Participant factors by NIH ordinal score 14

Supplementary Table 3. Ordinal regression results in multivariate analyses of NIH ordinal scores including analysis of IgG and IgA responses treated as separate and residual-to-each-other predictors 16

Supplementary Figure 3. Binary logistic regression outputs incorporating lessons from descriptive analysis and initial ordinal regression, informing the gradient boosting model 17

A. Distribution of observations and ROC curve in a pre-machine learning model 17

B. Visualization of how different values of the four antibody values contributed to prediction of an NIH Ordinal score of 7 in a pre-machine learning model 18

Supplementary Figure 4. Gradient boosting model incorporating days ill in contrast to main model with 14-day binary breakpoint 19

A. Compare with Figure 2 in the main manuscript 19

B. Exploration of Days of Illness and anti-N IgA relationships via SHAP curves 20

Supplementary Table 4. Quantitative bias analysis on antibody threshold selection from SHAP-curves 21

Acronyms employed in the manuscript and supplement

Acronym	Definition
AUC	Area under the curve in the context of a Receiver Operator Characteristic curve
BMI	body mass index
CI	confidence interval
COPD	Chronic Obstructive Pulmonary Disease
GOF	Goodness of Fit (particularly, for Hosmer-Lemeshow)
ICU	intensive care unit
Ig_	Immunoglobulin by isotype A or G, as indicated
kg	kilogram
MFI	median fluorescent intensity
N	Nucleocapsid (Nucleoprotein used synonymously)
n	number observed
NIH	National Institutes for Health (United States)
OR	odds ratio
RBD	Receptor Binding Domain
ROC	Receiver Operator Characteristic
SARS-CoV-2	Severe Acute Respiratory Syndrome Coronavirus-2
SD	standard deviation
yrs	years

Supplementary Table 1. Differences between antibody mean MFI for each NIH Ordinal score compared to those with a score of 4

	7 vs 4	6 vs 4	5 vs 4
**Anti-N IgA**	6,175 *(<0.001)*	3,302 *(0.03)*	3,118 *(0.085)*
**Anti-N IgG**	9,161 *(<0.001)*	-516 *(0.82)*	*1,780 (0.51)*
**Anti-RBD IgA**	3,297 *(<0.001)*	1,492 *(0.14)*	522 *(0.67)*
**Anti-RBD IgG**	7,544 *(0.0016)*	-1,876 *(0.43)*	-1,686 *(0.52)*

Note: This table supplements Figure 1 in the manuscript. Each cell compares the mean MFI for the given antibody level among those with the indicated NIH Ordinal score to those with a score of 4, the lowest observed in the cohort. Each value listed is mean MFI and *(p value by t-test)*.

Supplementary Figure 1. Bivariate relationships of co-factors with anti-SARS-CoV-2 MFI by target and isotype

A. Comparisons by age 65 years of age or older, or less than 65 years of age

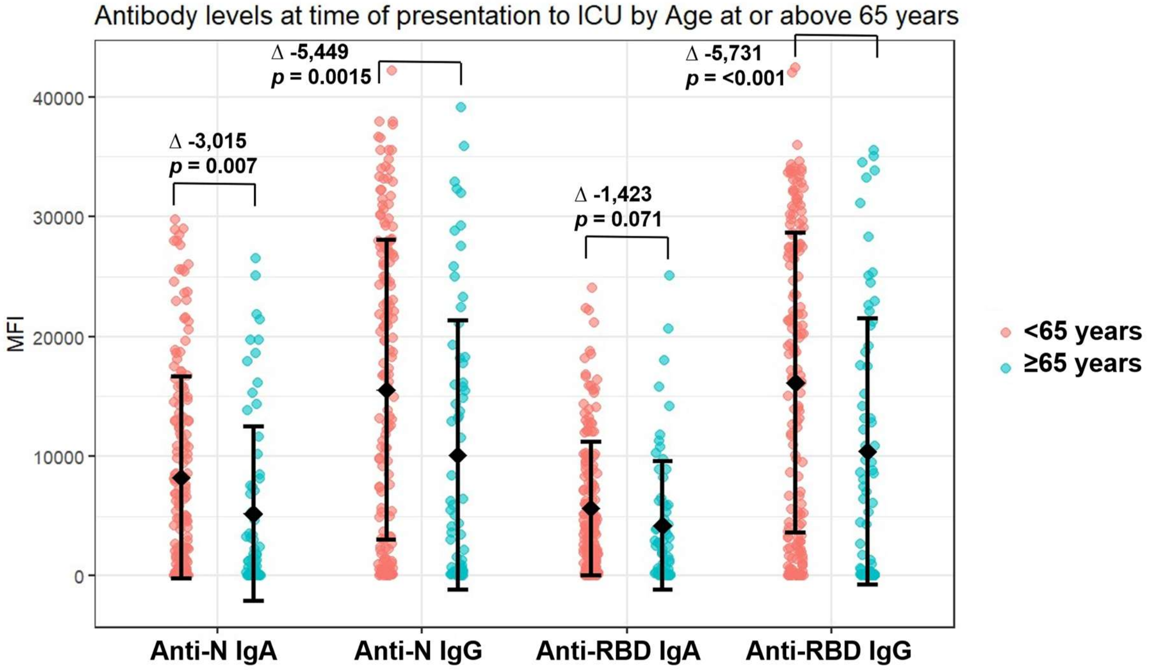

Note: Brackets demonstrate the difference in mean Median Fluorescent Intensity (MFI) of those age 65 year or older and those younger; *p* value by t-test.

B. Comparisons by whether or not the patient presented with a history of chronic pulmonary obstructive disease (COPD)

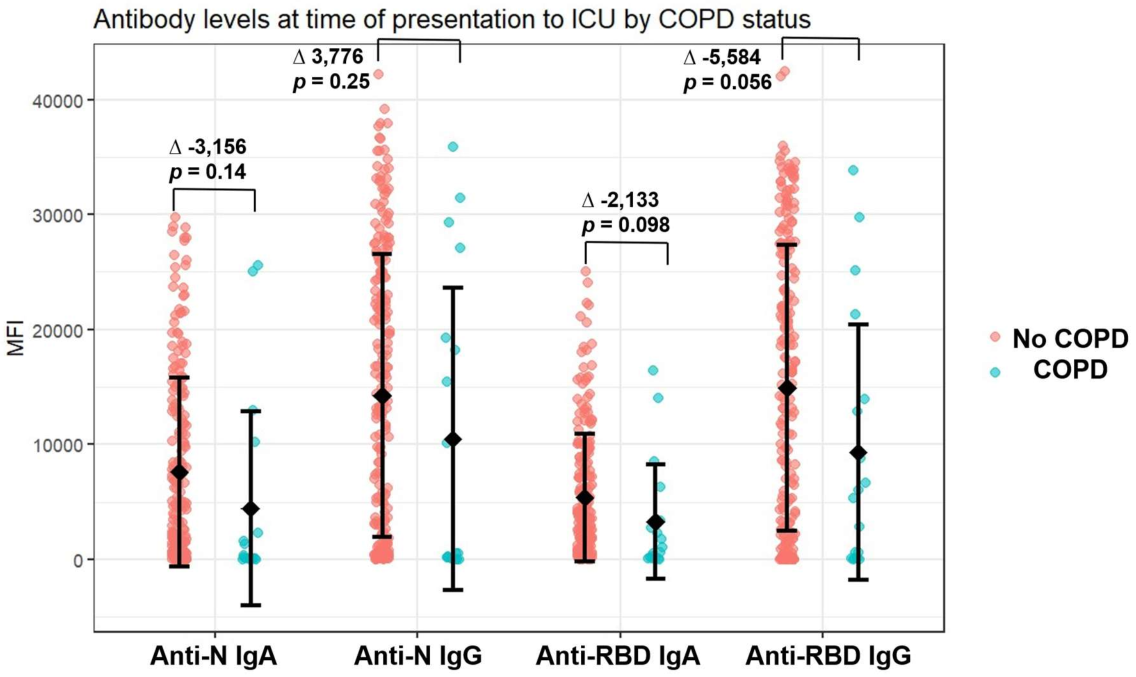

Note: Brackets demonstrate the difference in mean Median Fluorescent Intensity (MFI) of those with COPD and without; *p* value by t-test.

C. Comparisons by a body mass index (BMI) of 30 or higher, or less than 30

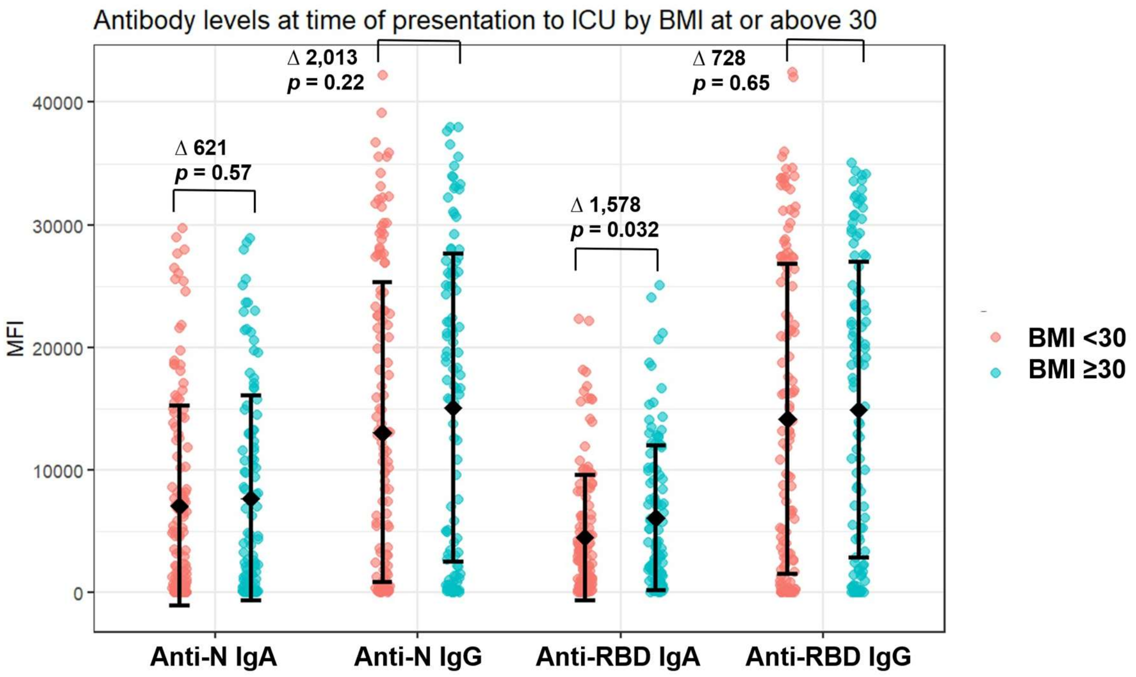

Note: Brackets demonstrate the difference in mean Median Fluorescent Intensity (MFI) of those with a BMI of 30 or greater and without; *p* value by t-test.

D. Comparisons by presentation at 14 days of illness or later, or presenting within 14 days

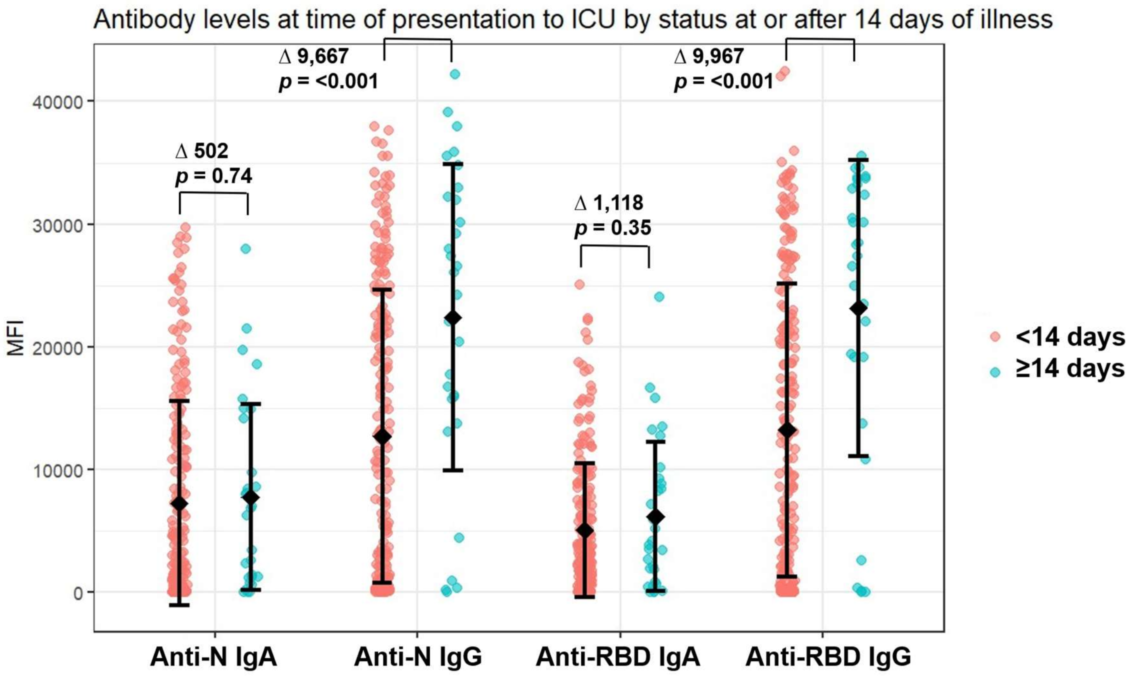

Note: Brackets demonstrate the difference in mean Median Fluorescent Intensity (MFI) of those who presented 14 days or more into their illness and not; *p* value by t-test.

Supplementary Figure 2. Bivariate distributions of log-transformed antibody targets coded by NIH ordinal score and host factors

A. Comparisons of antibody responses, with color coding by NIH Ordinal Score

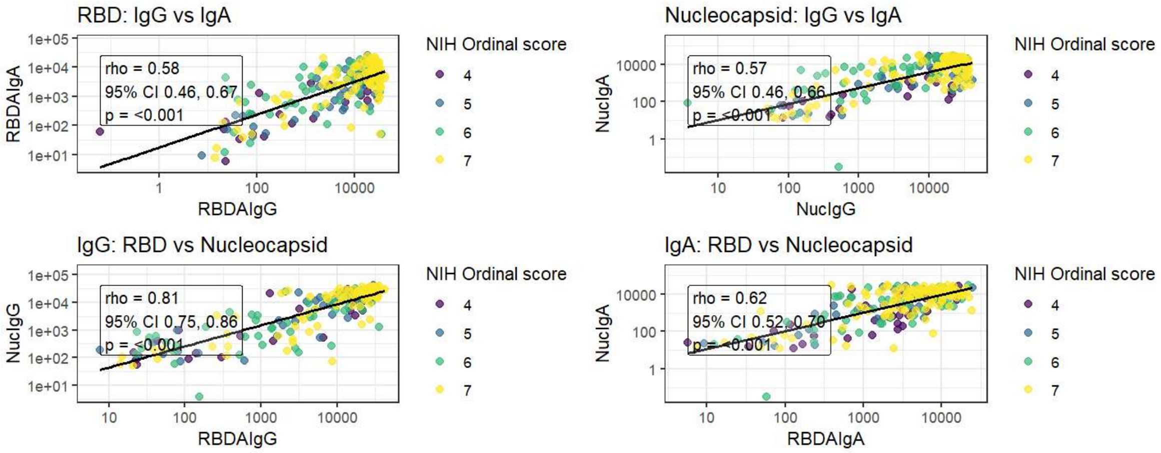

Note: These four graphs demonstrate the Spearman rho correlation analyses between IgG and IgA MFI against the same antigen, and within IgG and IgA against the two different antigen (RBD and N). Color coding is by NIH Ordinal score (ranging from 4 to 7 in this cohort) depicting COVID-19 disease severity. In addition to contributing to the descriptive analytic outputs, these were used to ensure that a range of values were represented when examining individual variability among candidate factors in order to avoid incorporating variables with equivalency in observation.

B. Comparisons of antibody responses, with color coding by age of 65 years of older, or less than 65 years

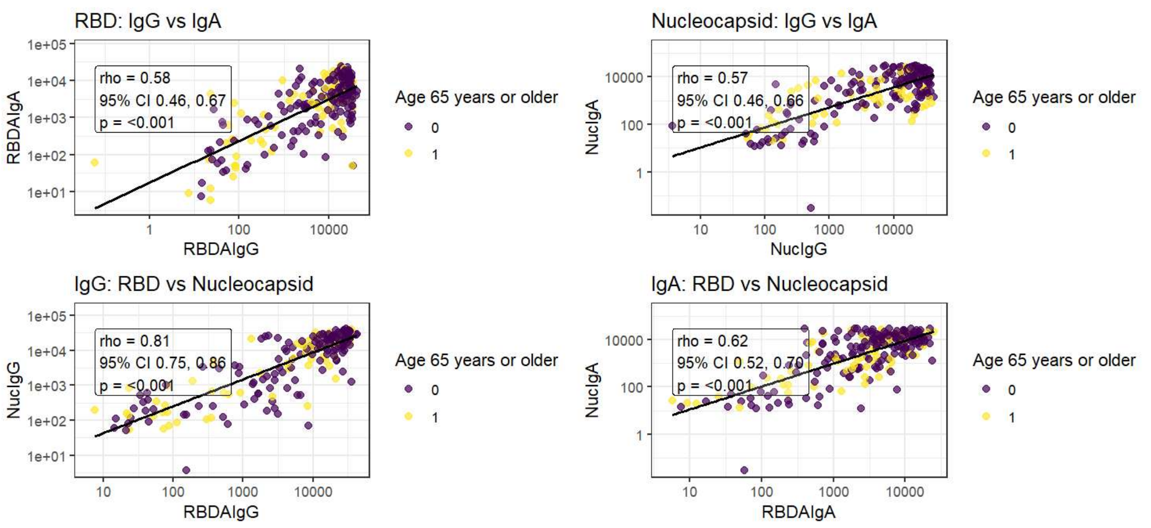

Note: These four graphs demonstrate the Spearman rho correlation analyses between IgG and IgA MFI against the same antigen, and within IgG and IgA against the two different antigens (RBD and N). Color coding is by whether participants were aged 65 years of older (a value of 1). In addition to contributing to the descriptive analytic outputs, these were used to ensure that a range of values were represented when examining individual variability among candidate factors in order to avoid incorporating variables with equivalency in observation.

C. Comparison of antibody responses, with color coding by whether or not the patient presented with a history of chronic pulmonary obstructive disease (COPD)

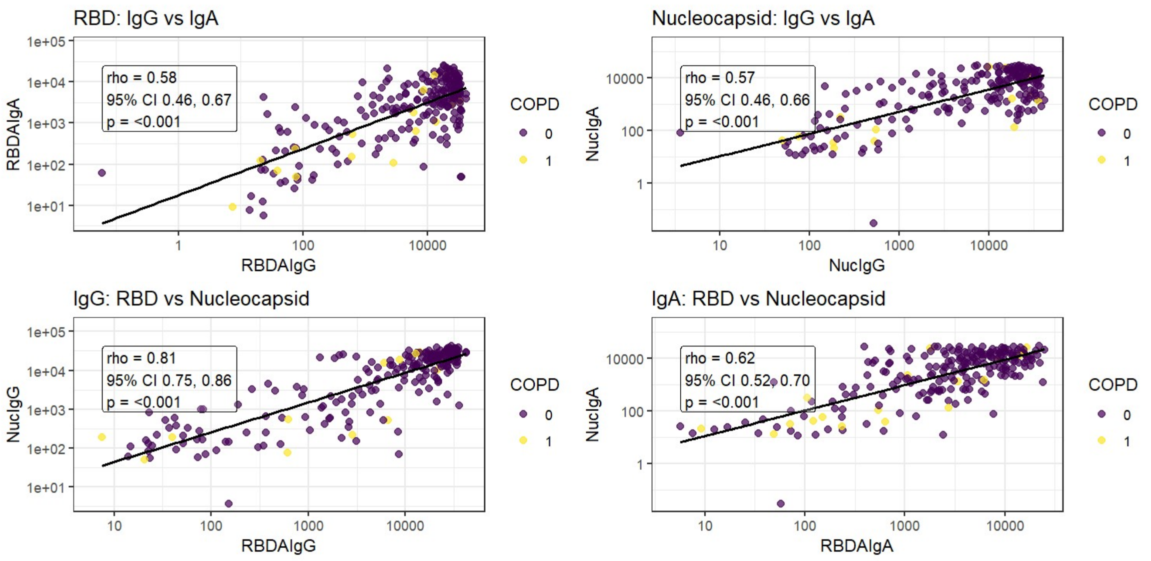

Note: These four graphs demonstrate the Spearman rho correlation analyses between IgG and IgA MFI against the same antigen, and within IgG and IgA against the two different antigens (RBD and N). Color coding is by whether participants had pre-existing COPD (a value of 1). In addition to contributing to the descriptive analytic outputs, these were used to ensure that a range of values were represented when examining individual variability among candidate factors in order to avoid incorporating variables with equivalency in observation.

D. Comparison of antibody responses, with color coding by a body mass index (BMI) of 30 or higher, or less than 30

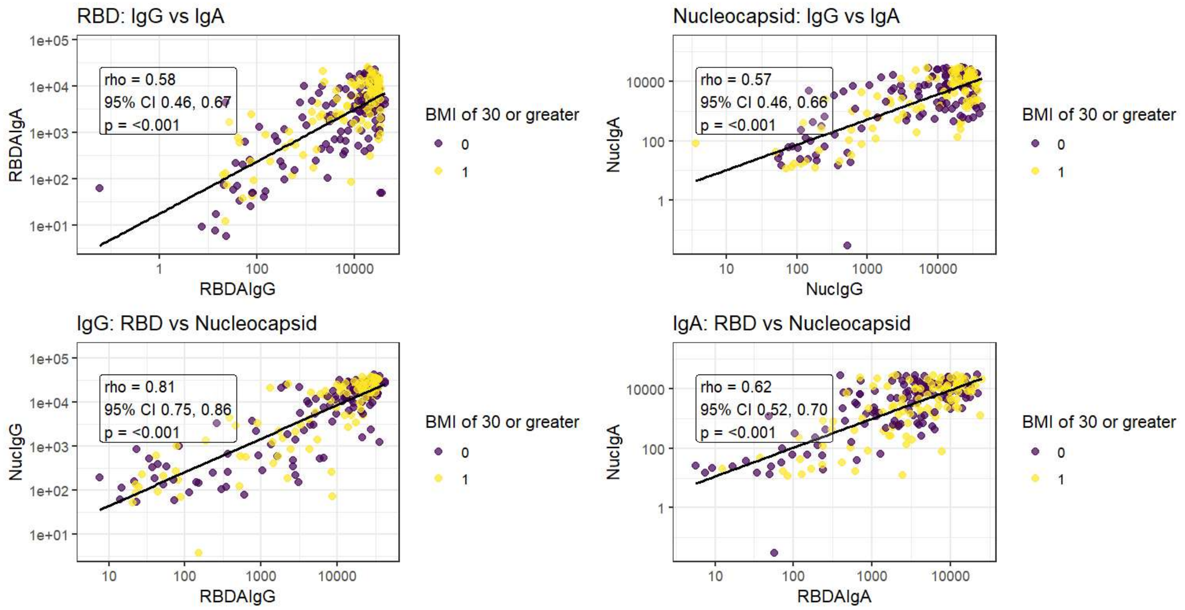

Note: These four graphs demonstrate the Spearman rho correlation analyses between IgG and IgA MFI against the same antigen, and within IgG and IgA against the two different antigens (RBD and N). Color coding is by whether participants had a BMI of 30 or higher (a value of 1). In addition to contributing to the descriptive analytic outputs, these were used to ensure that a range of values were represented when examining individual variability among candidate factors in order to avoid incorporating variables with equivalency in observation.

E. Comparison of antibody responses, with color coding by presentation at 14 days of illness or later, or presenting within 14 days

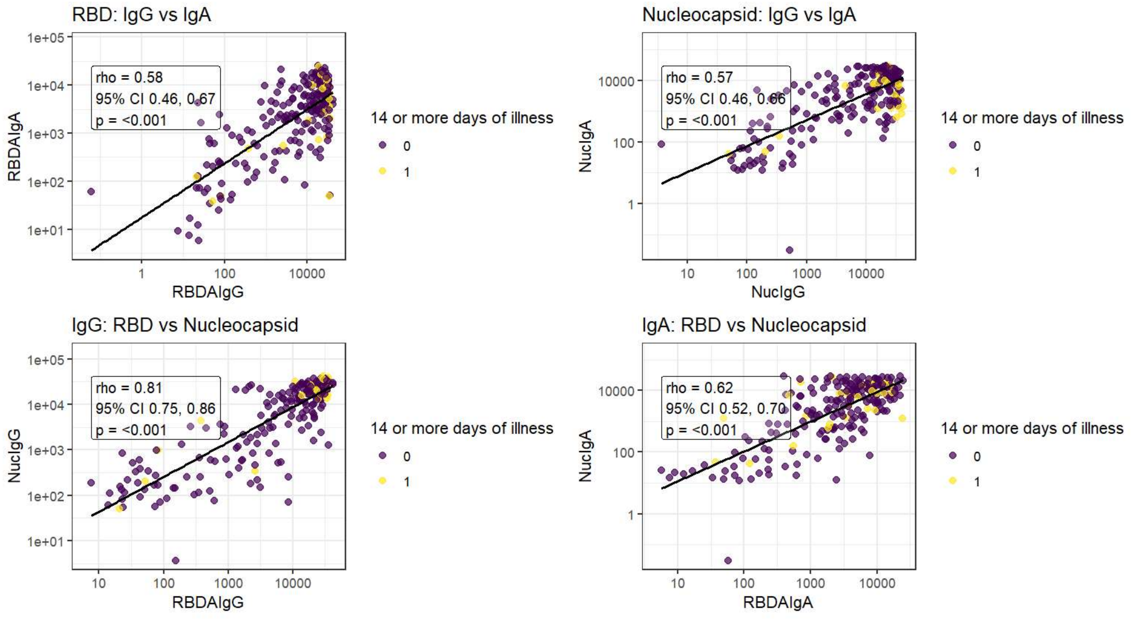

Note: These four graphs demonstrate the Spearman rho correlation analyses between IgG and IgA MFI against the same antigen, and within IgG and IgA against the two different antigens (RBD and N). Color coding is by whether participants were enrolled at 14 days or later in the course of their illness (a value of 1). In addition to contributing to the descriptive analytic outputs, these were used to ensure that a range of values were represented when examining individual variability among candidate factors in order to avoid incorporating variables with equivalency in observation.

Supplementary Table 2. Participant factors by NIH ordinal score

**Factors**	**4**<br>N = 31[1]	**5**<br>N = 37[1]	**6**<br>N = 62[1]	**7**<br>N = 108[1]	**p-value**[2]
**Age ≥ 65 yrs**					0.002
0	18 (58.1%)	24 (64.9%)	37 (59.7%)	90 (83.3%)	
1	13 (41.9%)	13 (35.1%)	25 (40.3%)	18 (16.7%)	
**COPD**					0.5
0	27 (87.1%)	34 (91.9%)	57 (91.9%)	102 (94.4%)	
1	4 (12.9%)	3 (8.1%)	5 (8.1%)	6 (5.6%)	
**BMI ≥ 30 kg/m²**					0.2
0	20 (64.5%)	23 (62.2%)	37 (59.7%)	51 (47.2%)	
1	11 (35.5%)	14 (37.8%)	25 (40.3%)	57 (52.8%)	
**Days ill ≥14**					<0.001
0	28 (90.3%)	36 (97.3%)	60 (96.8%)	84 (77.8%)	
1	3 (9.7%)	1 (2.7%)	2 (3.2%)	24 (22.2%)	
**Days ill** (linear)	4.3 (9.9)	2.6 (6.9)	3.5 (5.0)	8.7 (7.7)	<0.001
**Age** (linear)	59.6 (20.1)	57.5 (15.7)	58.4 (15.6)	51.2 (13.4)	0.002
**BMI** (linear)	27.5 (7.4)	30.7 (11.2)	30.7 (8.5)	32.6 (9.3)	0.008

Factors	**4** N = 31[1]	**5** N = 37[1]	**6** N = 62[1]	**7** N = 108[1]	**p-value**[2]
**Anti-RBD IgA**	3,257.1 (4,263.0)	3,778.7 (5,729.2)	4,748.8 (5,002.9)	6,554.5 (5,825.0)	<0.001
**Anti-RBD IgG**	11,819.1 (10,673.6)	10,133.2 (10,507.4)	9,942.8 (10,738.2)	19,363.1 (12,646.0)	<0.001
**Anti-N IgA**	3,205.5 (6,091.5)	6,324.0 (8,569.5)	6,507.6 (7,959.0)	9,380.2 (8,291.8)	<0.001
**Anti-N IgG**	9,664.5 (9,876.2)	11,444.8 (12,023.9)	9,148.8 (10,498.3)	18,826.0 (12,557.9)	<0.001

[1] n (%); Mean (SD)

[2] Pearson's Chi-squared test (categorical variables); Kruskal-Wallis rank sum test (linear variables)

Supplementary Table 3. Ordinal regression results in multivariate analyses of NIH Ordinal scores including analysis of IgG and IgA responses treated as separate and residual-to-each-other predictors

	**Ordinal results across 4-7**	**Score of 7 or not 7 (4-6)**		
**Factor**	**OR (95%CI)**	**OR**	**OR <14d**	**OR ≥14 d**
**Anti-N IgA**	1	1	1	1
**Anti-N IgA (*residual*)**		*No change when exchanged as residual to IgG*		
**Anti-N IgG**	1	1	1	1
**Anti-RBD IgA**	1	1	1	1
**Anti-RBD IgG**	1	1	1	1
**Anti-RBD IgG (*residual)***		*No change when exchanged as residual to IgA*		
**Age ≥ 65 yrs**	0.48 (0.28, 0.82)	0.48 (0.28, 0.82)	0.51 (0.29, 0.91)	0.35 (NA, NA)
**BMI ≥ 30**	1.56 (0.95, 2.6)	1.56 (0.95, 2.59)	1.56 (0.93, 2.64)	0.90 (NA, NA)
**COPD**	1.28 (0.5, 3.21)	0.78 (0.31, 1.97)	0.63 (0.24, 1.67)	NA
**Days ill ≥14**	4.45 (1.7, 13.4)	4.45 (1.61, 12.29)	NA	NA
**AUC**	*7 vs. 4-6:* 0.77 (0.71, 0.83)<br>*6 vs. 4-5:* 0.68 (0.61, 0.75)<br>*5 vs. 4:* 0.67 (0.58, 0.76)	0.92 (0.88, 0.96)	0.88 (0.83, 0.94)	0.99 (0.95, 1.0)

Note: OR denotes the odds ratio. In this set of analyses antibody variables were linear and so odds ratio outputs represent the impact of small incremental increases in MFI. The lower and upper limit 95% CI for each of the antibody levels odds ratios at three significant figures were 1.

Supplementary Figure 3. Binary logistic regression outputs incorporating lessons from descriptive analysis and initial ordinal regression, informing the gradient boosting model

A. Distribution of observations and ROC curve in a pre-machine learning model

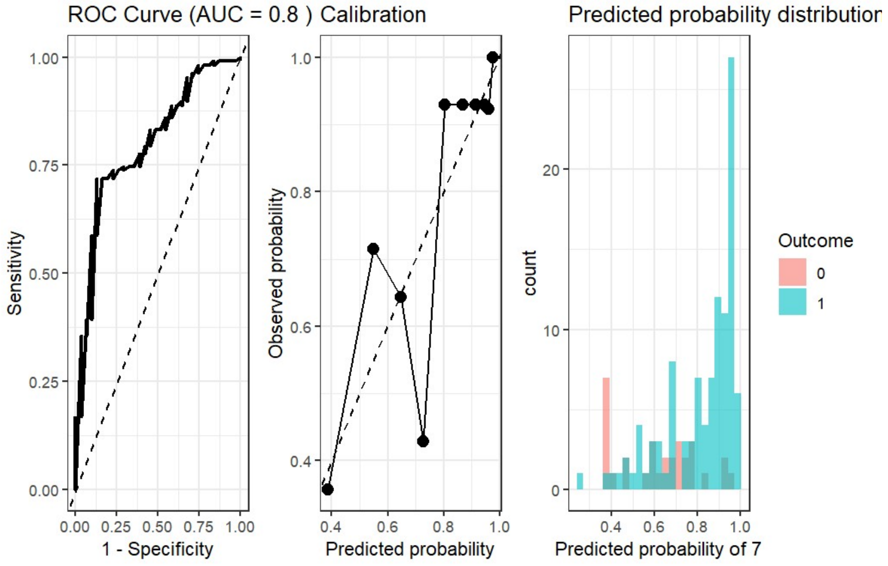

Note: This demonstrates that in a regression model assessing transformed candidate variables including IgG and IgA against RBD and N as well as COPD, BMI status, age 65 years or older, and 14 or more days of illness for the likelihood of having an NIH ordinal score of 7 versus 4, the AUC for the Receiver Operator Characteristic (ROC) curve is 0.8 with observations of varying probability informing an ordinal score of 7. This reinforced use of this set of variables as being relevant to the selected outcome and sufficiently distributed in probability gradient boosting machine learning might provide additional information than regressions regarding relative influence of variables.

B. Visualization of how different values of the four antibody values contributed to prediction of an NIH Ordinal score of 7 in a pre-machine learning model

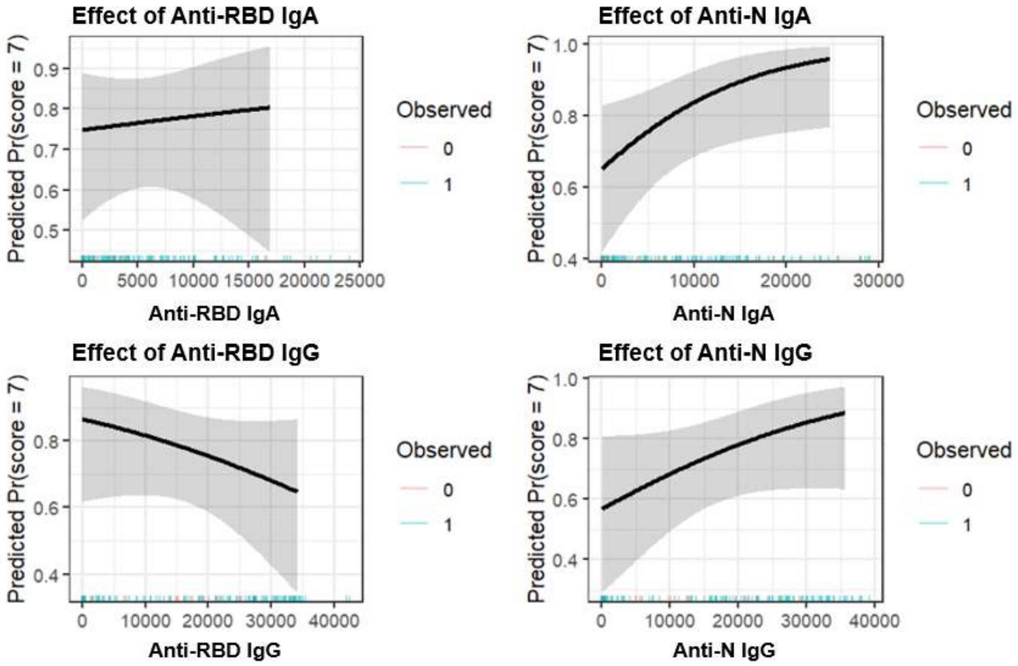

Note: This demonstrates that for each of the antibody targets, increasing MFI have continuous, unidirectional, and distinct patterns of association with prediction of an NIH Ordinal score of 7, reinforcing maintaining each of them in the gradient boosting machine learning. The green and orange vertical bars along the x-axes reflect antibody MFI values that were observed (a value of 1, green) versus imputed (a value of 0, orange). These distributions demonstrate that the behavior of these variables in the model were predominantly informed by observed rather than fitted values.

Supplementary Figure 4. Gradient boosting model incorporating days ill in contrast to main model with 14-day binary breakpoint

A. Compare with Figure 2 in the main manuscript

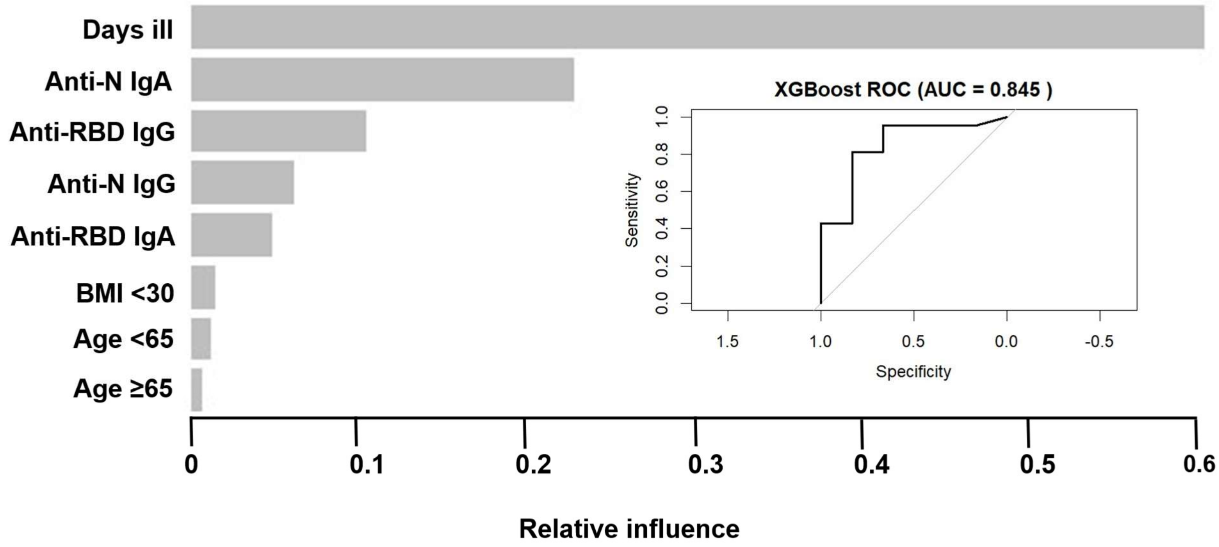

Note: Incorporating days of illness as a continuous variable in the gradient boosting machine learning mildly improves the AUC and re-orders the relative importance of days of illness, however, overall, the relative importance of anti-N IgA and anti-RBD IgG in relation to each other and the incorporated host factors is analogous to the results in the main analysis.

B. Exploration of Days of Illness and anti-N IgA relationships via SHAP curves

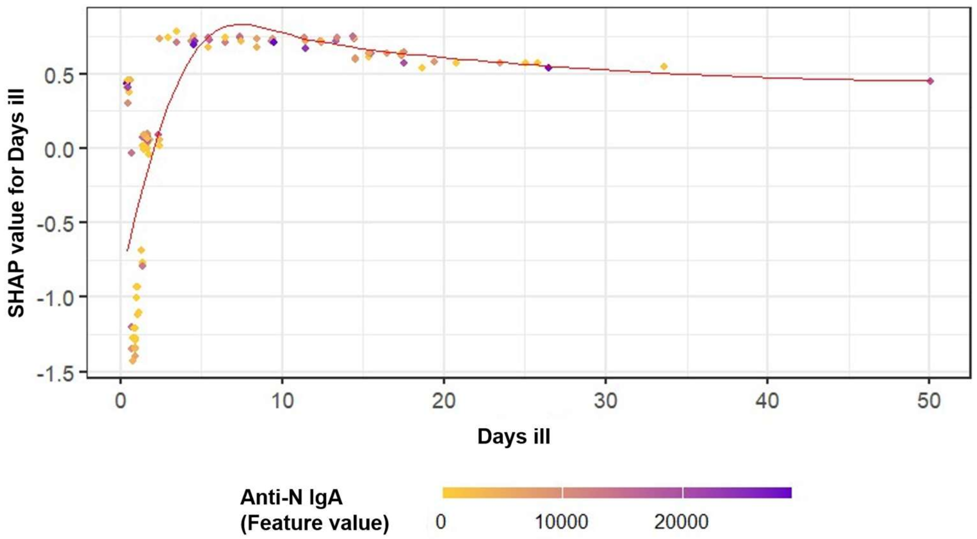

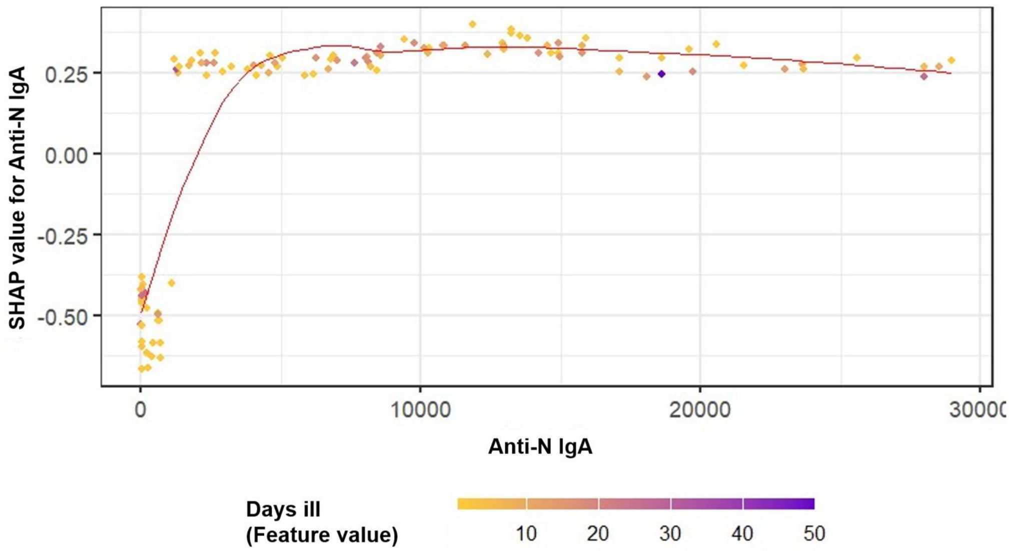

Note: In each curve, there are distinct populations (or sub-groups) of observations associated with different magnitudes and directions of relative influence. This suggests that if there is co-dependency between these variables, it is limited.

Supplementary Table 4. Quantitative bias analysis on antibody threshold selection from SHAP-curves

Odds ratios (95% CI) of individual factors in the multivariate model					
	**Anti-N IgA** 10,000 MFI **Anti-RBD IgG** 25,000 MFI	**Anti-N IgA** 5,000 MFI **Anti-RBD IgG** 25,000 MFI	**Anti-N IgA** 15,000 MFI **Anti-RBD IgG** 25,000 MFI	**Anti-N IgA** 10,000 MFI **Anti-RBD IgG** 20,000 MFI	**Anti-N IgA** 10,000 MFI **Anti-RBD IgG** 30,000 MFI
**Anti-N IgA**	4.3 (1.2-20.1)	3.4 (1-12.1)	2.4 (0.6-16.5)	4.3 (1.2-20.3)	3.4 (1-16.2)
**Anti-RBD IgG**	1 (0.3-3.8)	0.9 (0.2-3.4)	1.1 (0.3-4.2)	1 (0.3-3.6)	*
**Age ≥ 65 yrs**	0.4 (0.1-1.1)	0.4 (0.2-1.2)	0.4 (0.1-1)	0.4 (0.1-1.1)	0.4 (0.1-1)
**BMI ≥ 30**	1.5 (0.6-3.8)	1.7 (0.6-4.4)	1.5 (0.6-3.9)	1.5 (0.6-3.8)	1.6 (0.6-4.1)
**COPD**	0.6 (0.1-2.9)	0.7 (0.2-3.3)	0.5 (0.1-2.6)	0.6 (0.1-2.9)	0.6 (0.1-3.1)
**Days ill ≥14**	2.7 (0.7-13.6)	2.6 (0.7-13.3)	2.4 (0.7-11.7)	2.7 (0.7-13.5)	1.6 (0.4-8.3)
**Anti-RBD IgA**	1 (1-1)	1 (1-1)	1 (1-1)	1 (1-1)	1 (1-1)
**Anti-N IgG**	1 (1-1)	1 (1-1)	1 (1-1)	1 (1-1)	1 (1-1)
**Model performance**					
**AUC**	0.81 (0.73-0.89)	0.8 (0.72-0.89)	0.79 (0.71-0.87)	0.81 (0.73-0.89)	0.83 (0.75-0.9)
**Scaled Brier**	0.19	0.19	0.15	0.19	0.22
**GOF p-value**	0.74	0.3	0.28	0.83	0.89

Note: GOF refers to the Hosmer-Lemeshow goodness of fit test where a $p > 0.05$ is sought. The scaled Brier refers to the scaled Brier score for probabilistic prediction where values are between 0 and 1; lower but not 0 scores are preferred; used to compare performance of models—see https://pmc.ncbi.nlm.nih.gov/articles/PMC12818272.

*Uninterpretable, $p = 1$. 30,000 MFI is at the top of the linear range of this assay. Similar results were observed when using 27,500 MFI.
